# TIMP-1 Induces an EMT-Like Phenotypic Conversion in MDCK Cells Independent of Its MMP-Inhibitory Domain

**DOI:** 10.1371/journal.pone.0038773

**Published:** 2012-06-11

**Authors:** Young Suk Jung, Xu-Wen Liu, Rosemarie Chirco, Richard B. Warner, Rafael Fridman, Hyeong-Reh Choi Kim

**Affiliations:** Department of Pathology, Karmanos Cancer Institute, Wayne State University School of Medicine, Detroit, Michigan, United States of America; Technische Universität München, Germany

## Abstract

Matrix metalloproteinases (MMPs) and their endogenous inhibitors (TIMPs) regulate epithelial-mesenchymal transition (EMT) critical for the development of epithelial organs as well as cancer cell invasion. TIMP-1 is frequently overexpressed in several types of human cancers and serves as a prognostic marker. The present study investigates the roles of TIMP-1 on the EMT process and formation of the lumen-like structure in a 3D Matrigel culture of MDCK cells. We show that TIMP-1 overexpression effectively prevents cell polarization and acinar-like structure formation. TIMP-1 induces expression of the developmental EMT transcription factors such as SLUG, TWIST, ZEB1 and ZEB2, leading to downregulation of epithelial marker and upregulation of mesenchymal markers. Importantly, TIMP-1′s ability to induce the EMT-like process is independent of its MMP-inhibitory domain. To our surprise, TIMP-1 induces migratory and invasive properties in MDCK cells. Here, we present a novel finding that TIMP-1 signaling upregulates MT1-MMP and MMP-2 expression, and potentiates MT1-MMP activation of pro-MMP-2, contributing to tumor cell invasion. In spite of the fact that TIMP-1, as opposed to TIMP-2, does not interact with and inhibit MT1-MMP, TIMP-1 may act as a key regulator of MT1-MMP/MMP-2 axis. Collectively, our findings suggest a model in which TIMP-1 functions as a signaling molecule and also as an endogenous inhibitor of MMPs. This concept represents a paradigm shift in the current view of TIMP-1/MT1-MMP interactions and functions during cancer development/progression.

## Introduction

The epithelial-mesenchymal transition (EMT) is a morphogenetic process essential for all Metazoan embryogenesis. A partial EMT occurs during branching morphogenesis or tubulogenesis for the development of epithelial organs such as kidney, mammary gland, lung and salivary gland [1], [2], [3]. A phenotypic hallmark of EMT is that stationary epithelial cells, undergoing this biological process, acquire mesenchymal-like migratory properties. Importantly, studies suggest that carcinoma cells can re-activate the developmental EMT-like process during tumor cell invasion and metastasis [4]. Interestingly, genes involved in EMT are often conserved in different organs, throughout evolution, and as well during tumor progression. The three-dimensional (3D) Madin-Darby canine kidney (MDCK) cell culture is one of the most widely used experimental models to study the molecular mechanisms by which the EMT, lumen formation, and branching morphogenesis are regulated [5], [6], [7]. Accordingly, the MDCK model has been utilized to investigate the pathogenic EMT program that disrupts tightly regulated normal epithelial biology, contributing to human diseases such as organ fibrosis and carcinoma [8], [9], [10], [11].

Evidence suggests that matrix metalloproteinases (MMPs) and their endogenous inhibitors, tissue inhibitors of metalloproteinase (TIMPs), play critical roles during both developmental and pathological EMT. Increased expression of certain MMPs may function to disrupt cell-cell contact through E-cadherin [12]. As tumor progresses, tubular/ductal epithelial cells lose their epithelial properties and acquire mesenchymal-like features including loss of apical-basal cell polarity, acinar-like structures and cell-cell tight junction proteins as well as loss of the ability to invade through basement membranes and interstitial matrix. In an MDCK model, mitogen-activated protein kinase (MAPK)-regulated MMP-13 and TIMP-1 expression were involved in controlling partial-EMT and re-differentiation [11]. Also, overexpression of membrane-type 1-matrix metalloproteinase (MT1-MMP) resulted in differentiated and locally invading tumors [10]. Thus, deregulation of MMPs and/or TIMPs may result in the pathologic EMT process.

Among MMPs and TIMPs, TIMP-1 is frequently overexpressed and shown to serve as a prognostic marker in several types of human cancers including breast cancer, prostate cancer, lung cancer, melanoma, multiple myeloma, and glioblastoma [13], [14], [15], [16], [17], [18], [19], [20], [21]. This seemed at first counter-intuitive considering its prominent role in MMP inhibition, thereby suppressing matrix degradation necessary for tumor cell invasion. Importantly, we and others have demonstrated that TIMP-1 can function as a signaling molecule independent of its MMP-inhibitory domain in a variety of cell types, activating the cell survival program [22], [23], [24], [25], [26], [27], [28]. In breast epithelial cells, TIMP-1 interacts with the tetraspanin CD63 and subsequently activates an integrin β1 signaling complex, leading to activation of focal adhesion kinase (FAK), phosphoinositide 3-kinases (PI3K), Akt, and ERKs [22], [23], [24], [25]. In the MCF10A morphogenesis assay within a 3D Matrigel matrix, TIMP-1 signaling disrupts cell polarization and inhibits apoptosis in centrally located cells, thereby preventing the formation and maintenance of the hollow acinar-like structure [25].

The present study focused on the effects of TIMP-1 expression on lumen formation, EMT marker expression, and migratory/invasive properties of non-malignant immortalized Madin-Darby canine kidney (MDCK) cells. We report herein that while control MDCK cells cultured within a Matrigel matrix formed polarized acinar-like structures with hollow lumens, MDCK cells overexpressing TIMP-1 exhibited disrupted cell polarization and reduced cell death in the centers of the spheroids, and consequently, these cells were unable to undergo the acinar morphogenetic program. In addition, TIMP-1 induces expression of EMT transcription factors including SLUG, TWIST, ZEB1 and ZEB2, leading to downregulation of epithelial cadherin (E-cadherin) and upregulation of mesenchymal markers such as N-cadherin, fibronectin, and vimentin. Interestingly, contrary to the reported anti-migratory and anti-invasive properties of TIMP-1 [29], [30], [31], our data identify TIMP-1 as a promoter of MDCK cell migration and invasion. Lastly, we present a novel finding that TIMP-1 signaling induces MT1-MMP expression at both RNA and protein levels. Importantly, TIMP-1-induced EMT-like phenotypic changes and increased MT1-MMP expression are independent of TIMP-1′s MMP-inhibitory domain. Taken together, the present study provides a new molecular insight into the novel oncogenic activity of TIMP-1 during cancer progression.

## Materials and Methods

### Reagents and Antibodies

Mitomycin C, concanavalin A (ConA) were obtained from Sigma (St. Louis, MO). GM6001 was obtained from Calbiochem (San Diego, CA). Growth factor-reduced basement membrane Matrigel (GFR Matrigel) was purchased from BD Biosciences Discovery Labwares (Palo Alto, CA). Anti-TIMP-1 monoclonal antibody (Clone 102D1) was purchased from NeoMarkers, Inc. (Fremont, CA). Monoclonal antibodies against the N-terminus of MMP-2 and the catalytic domain of MT1-MMP were purchased from Millipore (Billerica, MA). Anti-E-cadherin mAb was purchased from BD transduction laboratories (San Jose, CA). Anti- β-actin mAb, anti-vimentin mAb, and peroxidase conjugated antibodies against mouse or rabbit IgG were purchased from Sigma (St. Louis, MO). Anti-GAPDH mAb was purchased from Santa-Cruz Biotechnology (Santa Cruz, CA). The FAK100 actin cytoskeleton kit was purchased from Chemicon International, Inc. (Temecula, CA).

### Cell Culture

Canine kidney epithelial MDCK cells were purchased from the American Type Tissue Collection (ATCC) and cultured at 37°C in a humidified incubator with 5% CO_2_, and DMEM media were supplemented with 10% fetal bovine serum, 2 mM glutamine, 100 units/ml penicillin, and 100 mg/ml streptomycin (Life Technologies Inc., Carlsbad, CA).

### Establishment of Human TIMP-1 and its Mutants Overexpressing MDCK Cells

Full-length TIMP-1 (amino acids 1–184) (T1) or the partial N-terminal and C-terminal domain (amino acids 66–184) of TIMP-1 (T1D) were amplified by PCR using a vector carrying hTIMP-1 cDNA from Open Biosystems (Huntsville, AL), and the various restriction enzyme sites were introduced respectively according to the target vector. The signal peptide was included in the constructs to maintain secretion. The pcDNA3.1 control, pcDNA3.1-T1 and pcDNA3.1-T1D vectors were transfected into MDCK cells using Lipofectamine 2000 (Invitrogen, Carlsbad, CA) according to the manufacturer’s protocol. Subsequently, cells were subjected to 400 µg/ml G418 antibiotic selection for 14 days and pooled for further analysis. Pooled populations expressing wild type TIMP-1 proteins are referred to as MDCK-T1 cells and pooled populations expressing N-terminal MMP inhibitory domain-deleted TIMP-1 (T1D) proteins are referred to as MDCK-T1D. Control cells transfected with pcDNA3.1 plasmid without an insert are referred to as MDCK-Neo cells. In order to detect T1D protein expression, the final PCR products for TIMP-1 and T1D were also cloned into p3XFLAG-CM-14 expression vectors. These expression vectors were also transfected into MDCK cells.

### Cell Proliferation Assay

Cell proliferation was assessed by MTT assay as well as trypan blue dye exclusion assay. 2000 cells were plated in a 96-well plate and cell viability was determined at the indicated time points by MTT assay as instructed by the manufacturer. Briefly, after incubation with MTT (0.5 mg/ml) for 4 h at 37°C, formazan precipitates formed by mitochondrial dehydrogenases in viable cells were extracted with acidic isopropanol. The absorbance of the converted dye was measured at a wavelength of 570 nm and the results were expressed as a percentage (%) compared to the number at time 0. For trypan blue dye exclusion assay, cells were seeded at 1×10^4^cells/60 mm plate. At the indicated time points, cells were stained with 0.4% Trypan blue dye solution (Sigma Chemical Co., St Louis, MO, USA) for 10 min and live cells, capable of excluding the dye with intact membranes, were counted using a hemocytometer. At least three independent experiments were performed for statistical analysis.

### MT1-MMP Knockdown

MDCK-T1 and MDCK-T1D cells were transfected with small interfering RNA (siRNA) targeting MT1-MMP or non-specific control siRNA using Lipofectamine 2000 according to manufacturer’s protocol. Two siRNA target sequences for canine MT1-MMP were synthesized by Dharmacon, Inc., Lafayette, CO, USA (set1, 5′- GGG AAC AAA UAC UGG AAA UUU -3′, 5′- AUU UCC AGU AUU UGU UCC CUU-3′ and set 2; 5′- GGG CUG AGA UCA AGG CCA AUU-3′, 5′- UUG GCC UUG AUC UCA GCC CUU-3′). After transfection, cells were maintained in DMEM medium with 5% FBS for 72 hours before pro-MMP-2 activation assay.

### Immunoblot Analysis

Cells were lysed with ice-cold RIPA buffer (0.05 M Tris–HCl, pH 7.4, 0.15 M NaCl, 0.25% deoxycholic acid, 1% NP-40, 1 mM EDTA) supplemented with 1 mM phenylmethylsulfonyl fluoride (PMSF), 2 mM sodium metavanadate (NaVO_3_), 1 mM sodium fluoride (NaF), and protease inhibitor cocktail. The protein concentration in the lysates was determined by the BCA procedure (Thermo Scientific). Equal amounts of protein samples in SDS sample buffer [1% SDS, 62.5 mM Tris-HCl (pH 6.8), 10% glycerol, 5% β-mercaptoethanol, and 0.05% bromophenol blue] were boiled for 5 min and subjected to reducing SDS-PAGE. After electrophoresis, the proteins were transferred to a nitrocellulose membrane. The membrane was blocked with 5% nonfat dry milk in 100 mM Tris-HCl (pH 7.5), 150 mM NaCl, and 0.2% Tween 20 (T-TBS) for 1 hr at room temperature. The membranes were incubated with T-TBS containing 5% milk and the primary antibodies. After three washes with T-TBS, the blot was incubated with the appropriate horseradish peroxidase-conjugated secondary antibodies. The antigen was detected using the Western Blot Chemiluminescence Reagent Plus (Perkin Elmer Life Sciences, Inc., Boston, MA), according to the manufacturer’s instruction.

### Semi-quantitative RT-PCR

mRNA was purified from cells using the RNeasy kit (Qiagen, Valencia, CA, USA). cDNA synthesis was performed with Superscript III First-Strand Synthesis System (Invitrogen, Carlsbad, CA, USA), followed by PCR using GoTaq Flexi DNA Polymerase (Promega, Madison, WI, USA). Forward and reverse canine specific forward and reverse primers used are as follows: E-Cadherin: 5′- AAAACCCACAGCCTCATGTC-3′, 5′- CACCTGGTCCTTGTTCTGGT-3′; N-cadherin: 5′-CCCAAGACAAGCGACTAAGC-3′, 5′-TGACAGCTGACCTGAGATGG-3′; Fibronectin: 5′-GGTTTCCCATTATGCCATTG-3′, 5′-TTCCAAGACATGTGCAGCTC-3′; Vimentin: 5′-CCGACAGGATGTTGACAATG-3′, 5′-TCAGAGAGGTCGGCAAACTT-3′; MMP2: GGATGCTGCCTTTAATTGGA, 5′-CGCACCCTTGAAGAAGTAGC-3′; MMP9: 5′-CAAACTCTACGGCTTCTGCC-3′, 5′-TGGCACCGATGAATGATCTA-3′; MT1-MMP: 5′- GGAGACAAGCACTGGGTGTT-3′, 5′- CATCACTGCCCATGAATGAC-3′; SLUG: 5′-AAGCAGTTGCACTGTGATGC-3′, 5′-GCAGTGAGGGCAAGAAAAAG-3′; SNAIL: 5′-CAAGGCCTTCAACTGCAAAT-3′, 5′-AAGGTTCGGGAACAGGTCTT-3′; TWIST: 5′-ACGAGCTGGACTCCAAGATG-3′, 5′- CACGCCCTGTTTCTTTGAAT-3′; ZEB-1: 5′-AGGCAGATGAAGCGAGATGT-3′, 5′-TCTGGTCCTCTTCAGGTGCT-3′; ZEB-2: 5′-ACGACATTCTGCAAGCCTCT-3′, 5′-GTGTCACTGCGCTGAAGGTA-3′; GAPDH: 5′-AACATCATCCCTGCTTCCAC-3′, 5′-GACCACCTGGTCCTCAGTGT-3′; TIMP-1 (common to human and canine TIMP-1): 5′- CACCAGAGAACCCACCATGGC-3′, 5′- CACTCTGCAGTTTGCAGG-3′.

### Gelatin Zymography

Gelatin zymography of conditioned media was performed using 10% Tris-glycine SDS-polyacrylamide gels containing 0.1% gelatin. Briefly, serum-free conditioned media were mixed with Laemmli sample buffer without reducing agents or heating and then subjected to 10% SDS-PAGE. The gels were then incubated (30 min at room temperature) in renaturating buffer (2.5% Triton X-l00 in H_2_O), rinsed in distilled H_2_0, and equilibrated for an additional 30 min in developing buffer (50 mM Tris buffer, pH 8.0, 200 mM NaC1, 5 mM CaCl_2_, and 0.02% Brij-35) followed by an incubation (16 h at 37°C) in fresh developing buffer. The gels were then stained with 0.5% Coomassie Blue R250 in a solution of 10% methanol and 5% acetic acid followed by destaining in 10% methanol and 5% acetic acid.

### Caspase Activity Assay

Cells were lysed in cell extract buffer [150 mmol/L NaCl, 50 mmol/L Tris-HCL (pH 7.5), 0.5 mmol/L EDTA, and 0.5% NP40], kept on ice for 30 minutes, and centrifuged at 15,000 Xg for 10 minutes. Fifty microliters of the cytosolic fraction were incubated for 60 minutes at 37°C in a total volume of 200 uL of caspase buffer [20 mmol/L HEPES (pH 7.5), 50 mmol/L NaCl, and 2.5 mmol/L DTT] containing 25 mol/L Ac-DEVD-AMC for caspase-3-like activity (BioSource International, Inc., Camarillo, CA). 7-Amino-4-methylcoumarin fluorescence, released by caspase activity was measured at 460 nm using 360 nm excitation wavelength on a Spectra Maxi Germini fluorescence plate reader (Molecular Devices, Menlo Park, CA). Caspase activity was normalized per microgram of protein as determined with a bicinchoninic acid protein assay reagent (Pierce).

### Fluorescence-Activated Cell Sorting (FACS) Analysis

Apoptotic cell number was determined by FACS analysis using violet ratiometric membrane asymmetry Probe, 4'-N,N-diethylamino-6-(N,N, N-dodecyl-methylamino-sulfopropyl)-methyl-3-hydroxyflavone (F2N12S) as recommended by the manufacturer’s protocol. Briefly, suspended cells were incubated for 5 min with 200 nM F2N12S solution and 1 uM SYTOX AADvanced™ dead cell stain solution. Apoptotic cells were analyzed by Becton Dickinson FACSscan flow cytometer and BD FACSDiva software.

### Cell Migration and Invasion Assay

For a scratch wound assay for cell migration, cells were grown to 90% confluence in complete medium in a 6-well plate and pre-treated with mitomycin C (25 ug/ml) for 30 min before an injury line was made using a 2-mm-wide plastic pipette tip. After rinsing with PBS, cells were allowed to migrate in serum-free media for 16 hr, and photographs were taken (X40) to assess cell motility. Cell migration was also assessed using 24-well Transwell chambers with polycarbonate filters (8 um pore size) (Corning Costar, Cambridge, MA). The cells were resuspended in 0.1% BSA containing DMEM medium and the lower compartments of the wells were filled with 750 ul of 1% FBS containing medium. 1×10^5^ cells were placed in the upper part of the Transwell and incubated for 16 hr. The *in vitro* invasive property of cells was assessed using a modified Boyden chamber assay. A total of 1×10^5^ cells were placed in the upper compartment of the invasion chamber (BD BioCoat Matrigel Invasion Chamber, BD Biosciences, Bedford, MA). For MMP inhibition experiments, 25 uM GM6001 was added to the cells in the upper compartment of the insert. The chambers were incubated for 16 hr at 37°C. The filters were then stained with crystal violet. Quantification of the migration or invasion assay was performed by counting the number of cells at the lower surface of the filters.

### MDCK Morphogenesis Assay in Three Dimensional (3D) Culture

Three-dimensional culture was carried out as previously described (Debnath et al., 2002; Mills et al., 2004). Assay medium (DMEM supplemented with 5% FBS, 50 U/ml penicillin, and 50 µg/ml streptomycin) containing 2% growth factor reduced Matrigel (GFR Matrigel) (BD Biosciences, Bedford, MA) was replaced every four days. At indicated time points, cells were washed four times with PBS containing 1 mM Ca^2+^ and Mg^2+^, fixed in 4% paraformaldehyde at room temperature for 20 min, and washed three times with PBS:Glycine buffer (130 mM NaCl, 7 mM Na_2_HPO_4_, 3.5 mM NaH_2_PO_4_, 100 mM glycine) for 10 min. Cells were permeabilized in 0.5% Triton X-100 in PBS for 10 min at 4°C. After three washes with PBS, cells were incubated with Rhodamine-conjugated phalloidin at room temperature for 30 min. After three washes with PBS, the coverslips were counterstained with DAPI (Roche applied Science, Indianapolis, IN) and mounted with anti-fade solution. Confocal immunofluorescence microscopic analysis was performed using the Zeiss LSM510 confocal microscopy system equipped with krypton-argon (488 and 568 lines) and ultraviolet (364 line) lasers.

## Results

**Figure 1:**
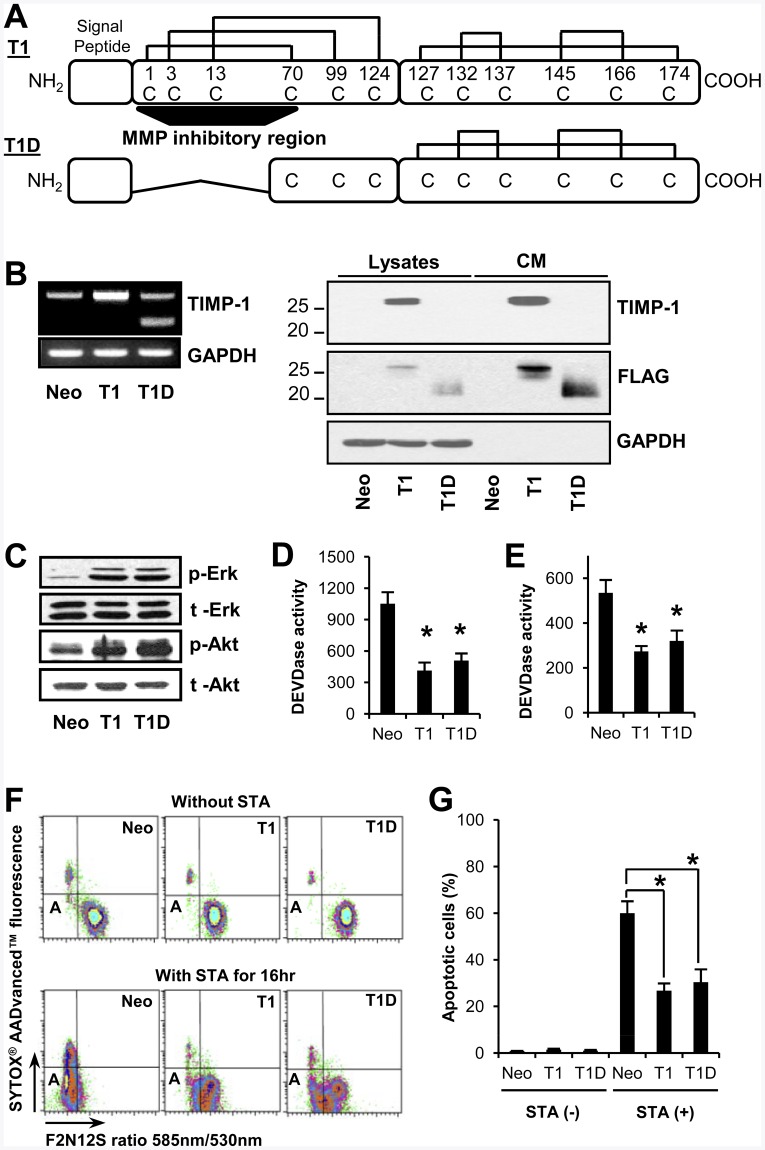
TIMP-1 induces cell survival signaling in MDCK cells, independent of its MMP-inhibitory domain. A. Schematic representation of the T1 and T1D proteins. The 12 cysteine (C) residues are linked to form six disulfide bonds; the 23 amino acids-long secretion signal sequence (amino acid 1–23) is indicated. A TIMP-1 mutant encoding the C-terminal and partial N-terminal (amino acid 66–184) regions of human T1 is indicated as TID. **B.** (Left Panel) RT-PCR analysis of TIMP-1 mRNA in MDCK-Neo, MDCK-T1 and MDCK-T1D cells using primers common to human and canine TIMP-1 mRNA. **B.** (Right Panel) Immunoblot analysis of TIMP-1 in cell lysates or conditioned media (CM) using anti-TIMP-1 Ab that recognizes the N-terminal domain of TIMP-1 (top panel) or anti-FLAG antibody (middle panel). **C.** Immunoblot analysis of Erk and Akt using MDCK-Neo, -T1, and –T1D cell lysates. **D-E.** DEVDase activity in MDCK-Neo, MDCK-T1, and MDCK-T1D cells upon treatment with 0.5 uM staurosporine for 4 hr (D) and serum-free culturing for 48 hr (E). DEVDase activity was normalized per microgram protein and each bar represents the mean ± s.d. of triplicates in three independent experiments. **F-G**. Apoptotic cells were quantified by FACS after F2N12S labeling and SYTOX AADvanced™ dead cell stain. (F) Representative FACS data without or with 0.5 µM staurosporine for 16 hr. (Upper left-dead cells; Lower left-apoptotic cells; Lower right-live cells). (G) Bar graph represents the mean percentages of apoptotic cells ± s.d. in the three independent experiment. Asterisk (*) indicates a *P* value <0.001 using a Paired T-test.

MDCK cells are non-malignant kidney epithelial cells that are known to activate an acinar-like morphogenetic program when cultured in 3-D conditions [32]. Moreover, MDCK cells can be reprogrammed to undergo an EMT under various stimuli [33], [34], [35]. Therefore, to examine the roles of TIMP-1 in regulation of EMT-like process and morphogenesis, we established MDCK cells engineered to overexpress wild-type TIMP-1 (referred to as MDCK-T1). To distinguish whether the TIMP-1 effects on EMT were mediated or not through inhibition of MMP activity, we also generated MDCK cells overexpressing TIMP-1 mutant in which the N-terminal MMP-inhibitory domain was deleted (referred to as MDCK-T1D), as also described elsewhere (Liu et al., manuscript under review) (Fig. 1A-B). As expected, purified recombinant T1D protein, lacking the binding site for active MMPs, completely lost its ability to inhibit MMP-2 and MMP-9 enzymatic activity (data not shown). As shown in Figure 1, ERKs and Akt, two signaling molecules that we previously identified as key mediators of the TIMP-1 survival pathway, were constitutively activated in both MDCK-T1 and MDCK-T1D cells. Consequently, both MDCK-T1 and MDCK-T1D cells were resistant to apoptosis induction when compared to control MDCK-Neo cells, as assessed by the caspase-like activity assay (Fig. 1D-E) and FACS analysis using a violet ratiometric membrane probe (Fig. 1F-G) upon staurosporine treatment or growth factor withdrawal. These results demonstrate that TIMP-1 activation of cell survival signaling in MDCK cells is independent of its MMP-inhibitory domain, consistent with previously findings [22], [23], [24], [25].

**Figure 2:**
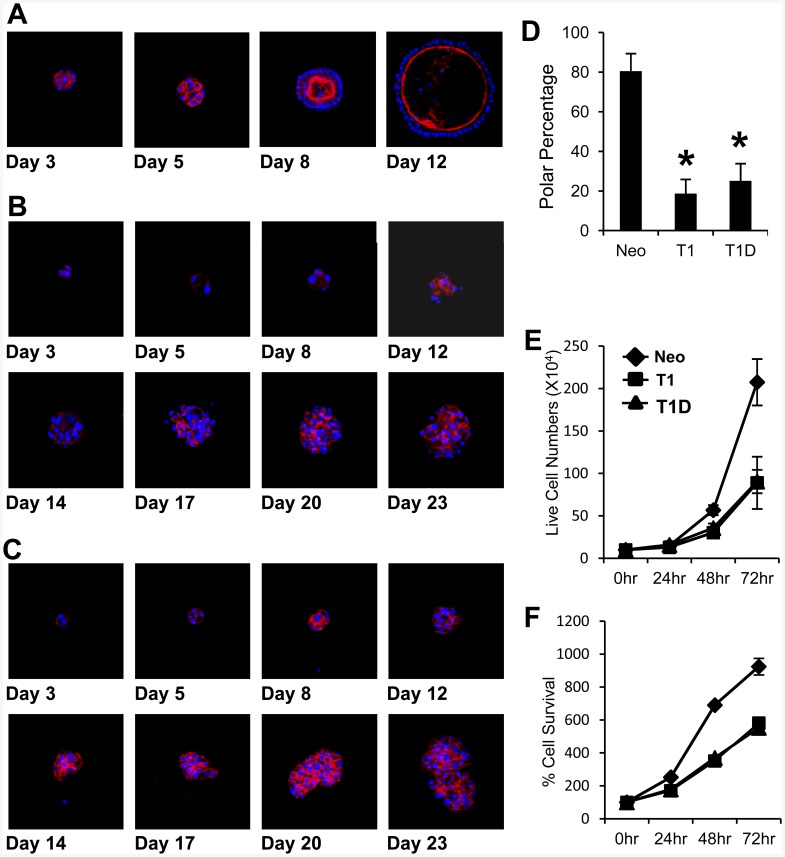
TIMP-1 signaling disrupts MDCK cell polarization and acini formation in a 3D Matrigel culture. Phalloidin staining (red) and blue-fluorescent nuclear staining with DAPI in MDCK-Neo (**A**), -T1 (**B**), and –T1D (**C**) cells in a 3D Matrigel culture for indicated periods. **D.** Percentage of polarized acini examined in cross sections through the middle of developing acini of MDCK-Neo at 12 days, and T1, T1D at 23 days. (More than 40 spheroids were analyzed for each condition from three independent experiments and the means + s.d. were shown). **E-F**. At the indicated time points, MDCK-Neo, T1, T1D cell proliferation was assessed by trypan blue exclusion assay (E) or MTT assay (F). (Shown are means + s.d of three independent experiments).

In a 3D Matrigel morphogenesis assay, it was previously shown that the outer layer of polarized MDCK cells in contact with the basement membrane survive, whereas the centrally located cells undergo cell death, a process that leads to the formation of a hollow acinar-like structure [32]. In agreement, MDCK cell polarization became evident within the cell cluster after 8 days in a 3D-Matrigel matrix, and the cells formed a hollow acinar-like structure within 12 days (Fig. 2A). In contrast, TIMP-1 overexpression prevented both polarization and cell death in centrally located cells, consequently inhibiting the formation of the hollow acinar structure (Fig. 2B). Interestingly, T1D expression prevented the formation of the acinar structure as effectively as the wild-type TIMP-1 (Fig. 2C). These results indicate that the ability of TIMP-1 to interfere with cell polarization and promote cell survival in the absence of cell contacts with basement membrane are independent of its MMP-inhibitory domain. As previously reported [36], TIMP-1 overexpression reduced the rate of cell proliferation as determined by MTT and trypan blue dye exclusion assays (Fig. 2E-F). It should be noted that TIMP-1 modulation of MDCK cell growth was independent of its MMP-inhibitory domain since the comparable effects were seen between wild-type TIMP-1 and T1D overexpression. To exclude the possibility that the lack of MDCK-T1 and MDCK-T1D cell polarization up to day 12 merely reflects delayed morphogenesis due to reduced cell proliferation rates, the morphogenesis assay was continued for 23 days (Fig. 2B and 2C). Under these conditions, ∼80% of MDCK-Neo cells displayed a well-polarized, hollow acinar-structure, whereas only ∼20% of MDCK-T1 and MDCK-T1D cells formed polarized spheroids (Fig. 2D), demonstrating a role for TIMP-1 in disrupting MDCK cell morphogenesis.

**Figure 3:**
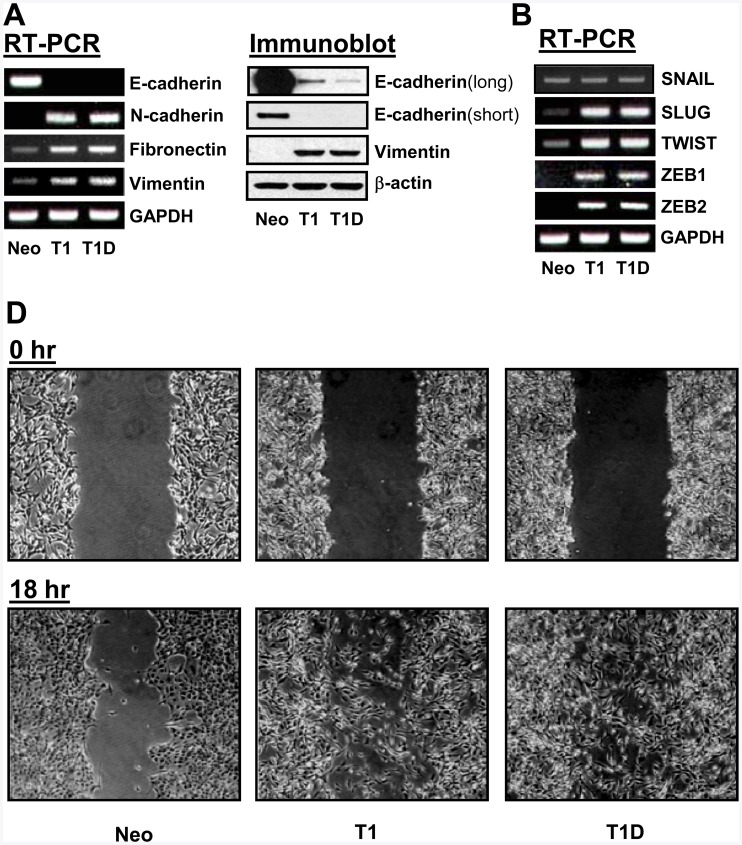
TIMP-1 regulates EMT markers and induces motility of MDCK cells, independent of its MMP-inhibitory domain. A. RT-PCR analysis (left panel) of epithelial marker E-cadherin and mesenchymal markers N-cadherin, Fibronectin, and Vimentin. Immunoblot analysis (right panel) of E-cadherin (after long and short exposures) and vimentin in MDCK-Neo, -T1, and –T1D cells. **B.** RT-PCR analysis of EMT transcriptional factors SNAIL, SLUG, TWIST, ZEB1 and ZEB2. **C.** A scratch migration assay was performed in MDCK-Neo, -T1 and T1D cells for 18 h.

**Figure 4:**
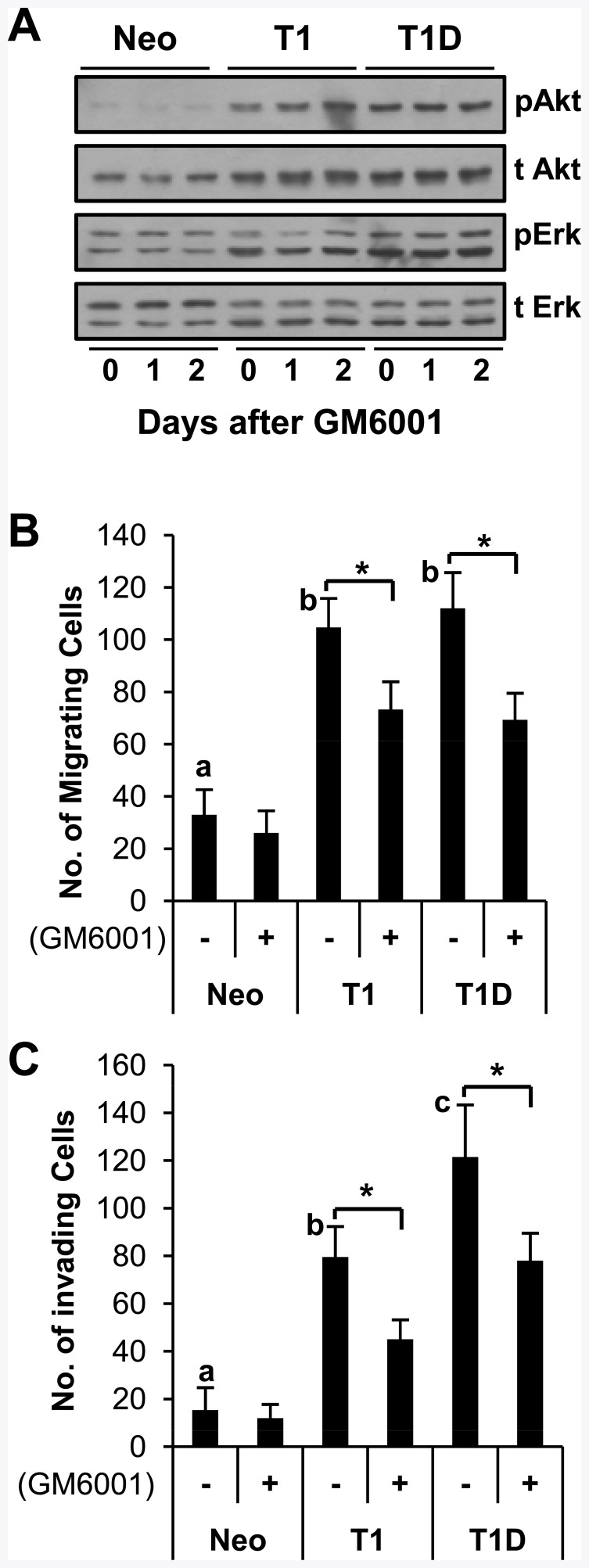
TIMP-1, but not GM6001 treatment, enhances MDCK cell migration and invasion. A. MDCK-Neo, -T1, and –T1D cells were incubated with 25 µM GM6001 and cell lysates were collected at the indicated time points, followed by immunoblotting analysis for Akt and ERKs. **B.** Cell migration was determined using a Transwell chamber assay for 18 h. The total number of cells that migrated to the lower side of the filter were counted using microscopy at 100X. **C.** Cell invasion was assessed using a BioCoat Matrigel Invasion Chamber for 16 h. The total number of cells that invaded to the lower side of the filter were counted using microscopy at 100X. The experiment was done in triplicates and the data is representative of three independent experiments. Each bar represents the mean ± s.d. Means with different letters (a, b, c) are significantly different from one another at *P* value <0.05 (ANOVA followed by Newman-Keuls test) and asterisk (*) indicates a *P* value <0.01 using a paired T-test.

Loss of epithelial cell polarity is often accompanied with changes in EMT marker gene expression. Thus, we examined the roles of TIMP-1 in the regulation of established EMT markers. As shown in Figure 3, TIMP-1 effectively downregulated E-cadherin expression and upregulated the mesenchymal markers N-cadherin, fibronectin, and vimentin (Fig. 3A). Interestingly, TIMP-1 upregulated the expression of the EMT transcription factors SLUG, TWIST, ZEB1 and ZEB2, while it had little effect on SNAIL expression (Fig. 3B). When we examined the effects of TIMP-1 on cell migration, which is a functional hallmark of EMT, both MDCK-T1 and MDCK-T1D cells displayed a more motile phenotype, as assessed by a scratch wound assay, when compared to MDCK-Neo cells (Fig. 3C). We also performed a modified Boyden chamber migration assay to better quantitate the effects of TIMP-1 and T1D expression on MDCK cell migration. As shown in Figure 4B, 3–4 fold increases in cell migration were detected in MDCK-T1 and MDCK-T1D cells compared to MDCK-Neo cells (T1, T1 D *vs.* Neo in the absence of GM6001 treatment). These results demonstrate that the ability of TIMP-1 to induce EMT in MDCK cells is not mediated by its MMP inhibitory domain and thus is independent of its ability to inhibit MMP activity. To further evaluate a potential contribution of MMP inhibition to the TIMP-1-mediated EMT process, cells were treated with GM6001, a broad-spectrum MMP inhibitor, and examined for activation of Akt and Erks and for cell motility. As shown in Figure 4A, neither Akt nor Erks activation was altered in MDCK-Neo cells after GM6001 treatment. These results are consistent with the notion, and previous finding [22], [23], [24], [25], that TIMP-1-induced survival signaling is independent of its MMP-inhibitory function. Interestingly, however, motility of MDCK-T1 and MDCK-T1D cells appears to be slightly reduced in the presence of GM6001 (Fig. 4B), suggesting the potential involvement of MMP activity for induction of cell motility.

**Figure 5:**
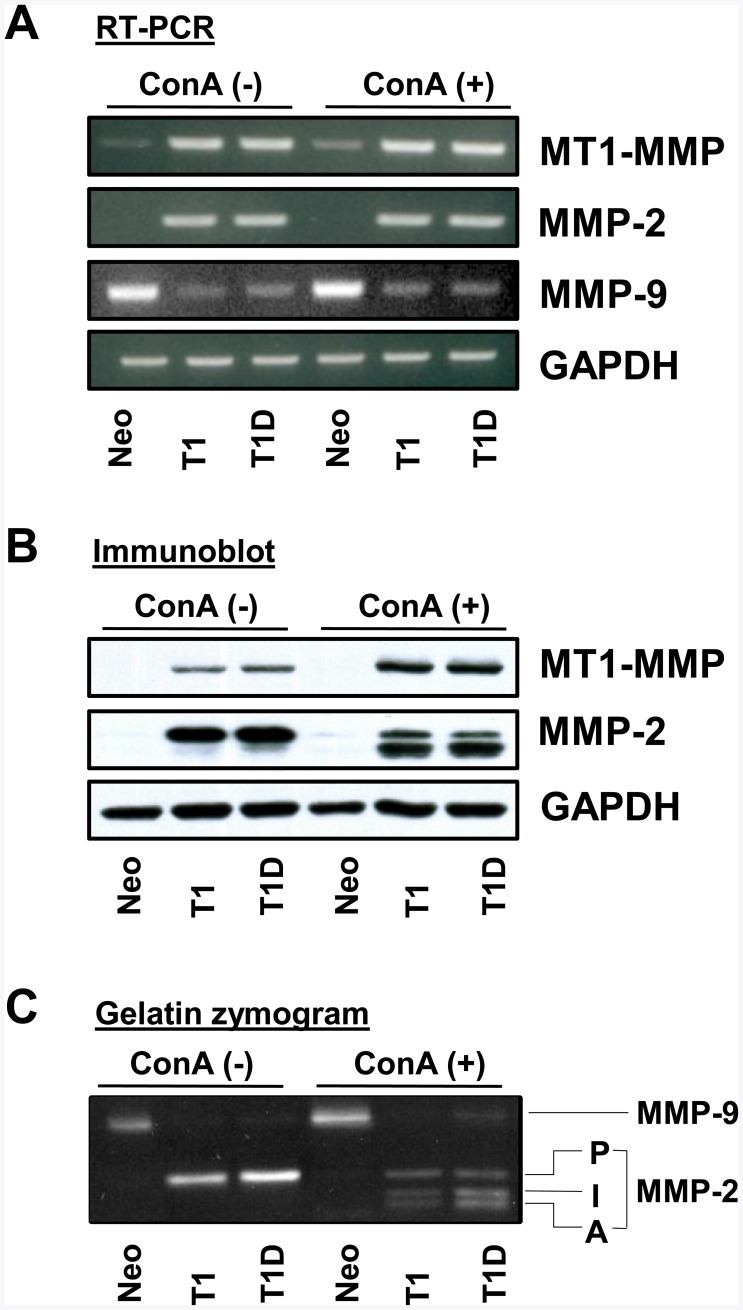
TIMP-1, independent of its MMP-inhibitory domain, upregulates the MT1-MMP/MMP-2 axis in MDCK cells. MDCK-Neo, -T1, and T1D cells were untreated (-) or treated (+) with ConA for 18 hr. **A.** MT1-MMP, MMP-2, and MMP-9 RNA levels were determined by semi-quantitative RT-PCR. **B.** Cell lysates and conditioned media were resolved by reducing 10% SDS-PAGE followed by immunoblot analysis of MT1-MMP and MMP-2. **C.** Conditioned media were analyzed by gelatin zymography. P, pro-MMP-2: I, intermediate form of MMP-2: A, active MMP-2.

EMT has been linked to enhanced tumor cell invasion [4]. Therefore, we compared the invasive capacity of MDCK-Neo, MDCK-T1 and MDCK-T1D cells using a Matrigel invasion assay. As shown in Figure 4C, both MDCK-T1 and MDCK-T1D cells readily invaded through Matrigel, when compared to MDCK-Neo cells, with MDCK-T1D cells exhibiting the highest invasive activity. Interestingly, TIMP-1- and T1D-induced MDCK invasion of Matrigel was partially sensitive to GM6001, suggesting a role for metalloproteinase activity in this process. Based on these results, we hypothesized that the enhanced invasiveness of both MDCK-T1 and MDCK-T1D cells is partly dependent on TIMP-1 signaling-mediated upregulation of matrix metalloproteinase that is less sensitive to TIMP-1 for its enzymatic inhibition, such as MT1-MMP [37], [38], [39]. Indeed, analyses of MT1-MMP expression revealed increased MT1-MMP mRNA and protein in MDCK-T1 and MDCK-T1D cells when compared to MDCK-Neo cells (Fig. 5A and 5B, lanes 1–3). In addition, MDCK-T1 and MDCK-T1D cells exhibited higher levels of MMP-2 but reduced levels of MMP-9 mRNA (Fig. 5A, lanes 1–3). Consistently, gelatin zymographic analysis showed increased MMP-2 and decreased MMP-9 protein expression in MDCK-T1 and MDCK-T1D cells when compared to MDCK-Neo cells (Fig. 5C, top band, lanes 1–3). Increased MMP-2 expression was also confirmed by immunoblot analysis (Fig. 5B, middle panel, lanes 1–3). MT1-MMP is a major activator of pro-MMP-2 [40]. To evaluate the functional significance of TIMP-1 upregulation of MT1-MMP, MDCK-T1, MDCK-T1D and MDCK-Neo cells were treated with lectin ConA, since MT1-MMP-dependent pro-MMP-2 activation requires stimulation with the lectin ConA [41]. Consistent with the higher levels of MT1-MMP expression in MDCK-T1 and MDCK-T1D cells, ConA treatment of these cells resulted in appearance of active MMP-2 (∼62 kDa) in the supernatant, as determined by immunoblot analyses and zymography (Fig. 5B and 5C).

**Figure 6:**
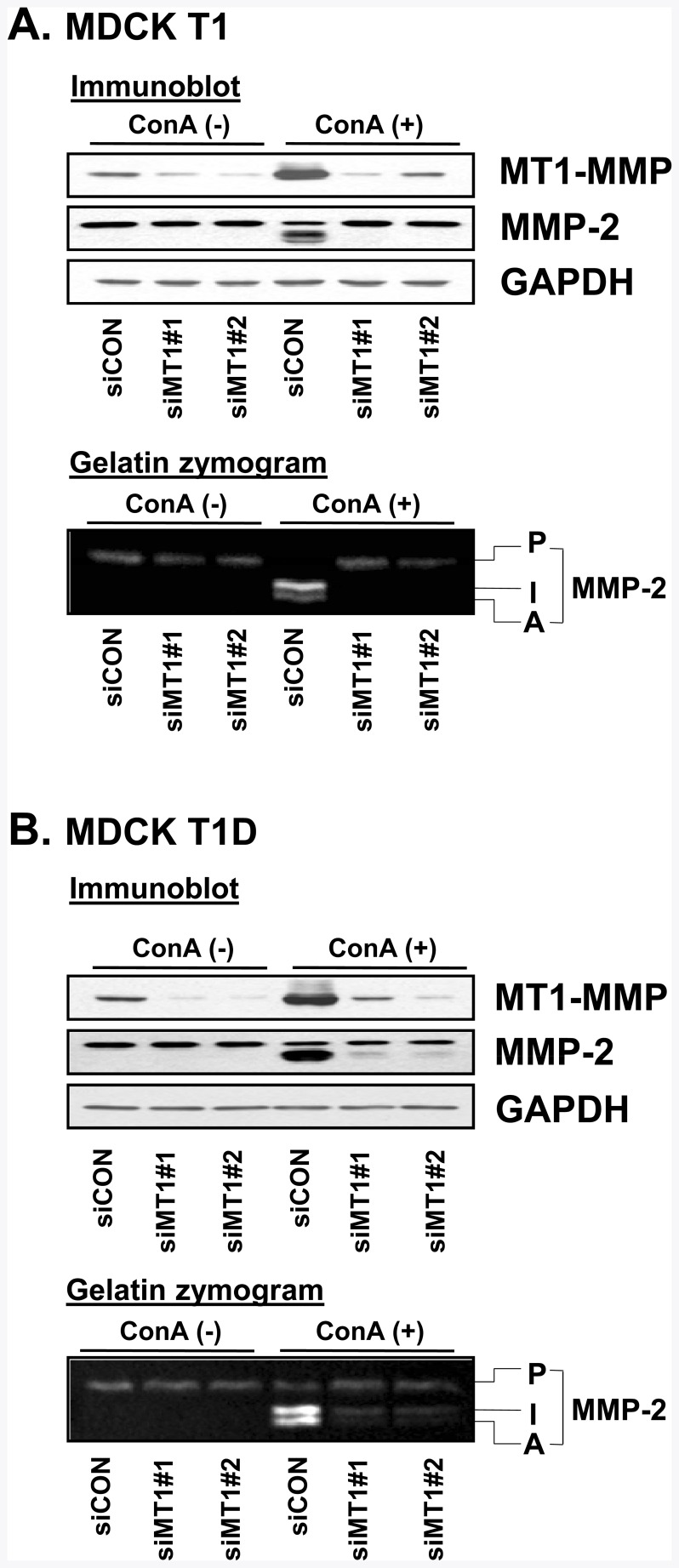
Knockdown of MT1-MMP abrogated MMP-2 activation in the MDCK-T1, T1D cells. MDCK-T1 (A) and MDCK-T1D (B) cells in the absence or presence of control or MT1-MMP siRNAs were untreated (-) or treated (+) with ConA for 18 hr. The protein levels of MT1-MMP in cell lysates and MMP-2 in conditioned media were determined by immunoblot analysis. Conditioned media were analyzed by gelatin zymography. P, pro-MMP-2: I, intermediate form of MMP-2: A, active MMP-2.

To determine the functional significance of MT1-MMP for increased MMP-2 activation in MDCK-T1 and MDCK-T1D cells, MT1-MMP expression was inhibited in these cells using 2 different siRNAs targeted to MT1-MMP. Immunoblot analysis confirmed significant downregulation of MT1-MMP expression in both MDCK-T1 and MDCK-T1D cells (top panels in Fig. 6A and 6B, respectively) upon siRNA transfection. Importantly, MT1-MMP knockdown resulted in drastic inhibition of MMP-2 activation upon ConA treatment. It should be noted that MT1-MMP knockdown had little effect on the expression levels of MMP-2 and MMP-9.

**Figure 7:**
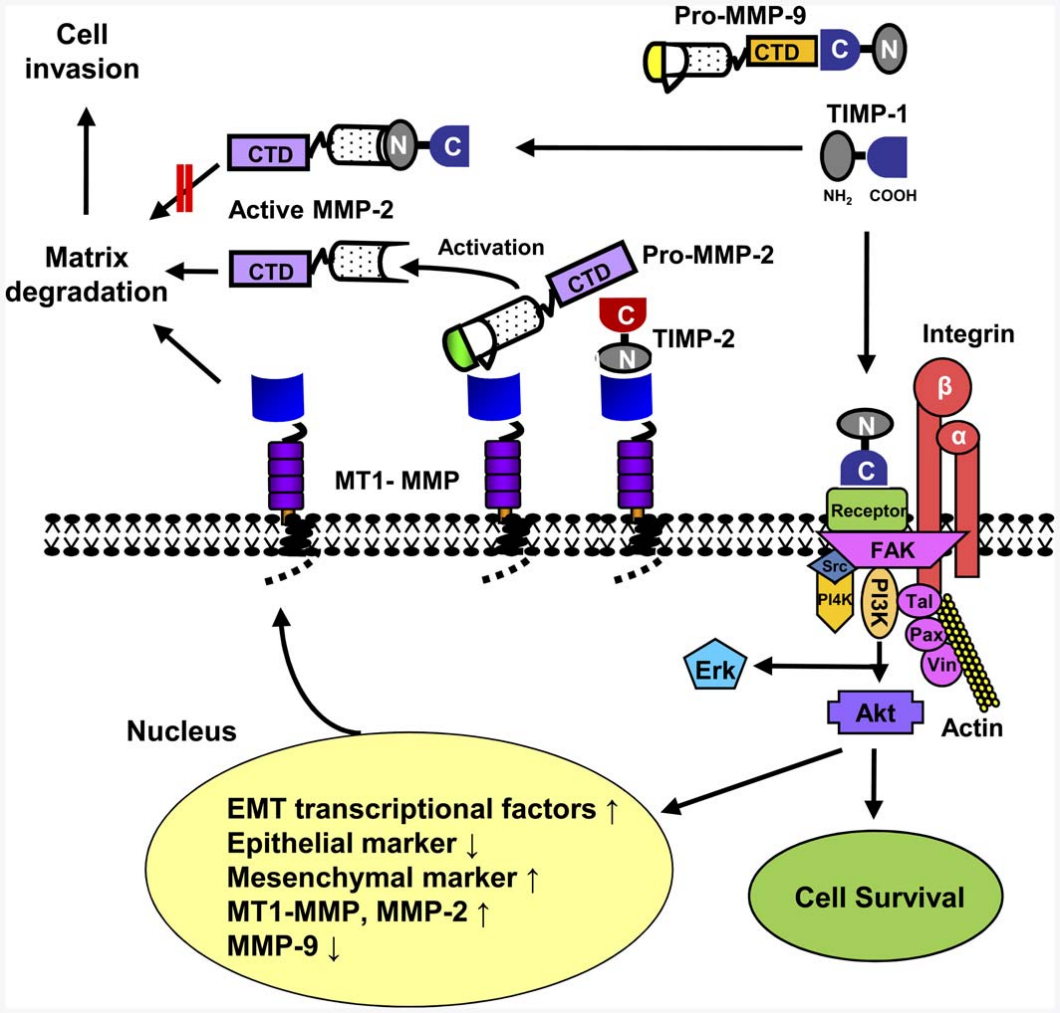
A working model of TIMP-1 regulation of cell survival and MT1-MMP/MMP2 axis during EMT. TIMP-1 interacts with cell surface protein(s) via its C-terminal domain, resulting in activation of cell survival signaling pathways and regulation of gene expression. Increased MT1-MMP expression on the cell surface promotes MMP-2 activation. The pro-MMP-9 protein can interact with the C-terminal domain of TIMP-1, and thereby may reduce TIMP-1′s signaling capacity. TIMP-1 signaling-mediated downregulation of MMP-9 expression allows TIMP-1, free of pro-MMP-9, to further promote cell signaling, a positive feedback signal amplification.

Taken together, we propose that TIMP-1 signaling, independent of its MMP-inhibitory domain, regulates expression of a set of genes such as EMT transcription factors, epithelial/mesenchymal markers, MT1-MMP, MMP-2 and MMP-9. We also propose that increased MT1-MMP expression on the cell surface plays a critical role in MMP-2 activation as depicted in Figure 7.

## Discussion

The present study demonstrated a novel function of TIMP-1 in inducing EMT transcription factor expression and activating the MT1-MMP/MMP-2 axis, leading to induction of the EMT-like process in MDCK cells. Taking these results together with our previous findings, we propose a working model for pleiotropic activities of TIMP-1 as depicted in Figure 7. The C-terminal domain of TIMP-1 interacts with a cell surface receptor, which in turn activates signaling molecules such as FAK, PI3K, Akt, and MAPK, thereby inducing cell survival and EMT [22], [23], [24], [25]. In human breast epithelial MCF10A cells, we previously identified the tetraspanin, CD63, as a cell surface receptor for TIMP-1, and TIMP-1 activates an integrin β1 signaling complex in a CD63-dependent manner [25]. Since the canine counterpart of the human CD63 gene has not been characterized and the commercially available antibodies against CD63 failed to detect canine proteins, we could not investigate the involvement of CD63 in TIMP-1-mediated EMT process in the MDCK model. Nonetheless, similar to TIMP-1 interactions with CD63 and subsequent signal transduction in breast epithelial cells, the N-terminal MMP-inhibitory domain was not necessary for TIMP-1 signaling in MDCK cells.

Our novel finding of TIMP-1 regulation of MT1-MMP, MMP-2, and MMP-9 expression may bring a paradigm shift in regards to our understanding of TIMP-1′s function as an endogenous regulator of MMPs. The C-terminal part of TIMP-1functions as a signaling molecule and regulates the MT1-MMP/MMP-2 axis, whereas the N-terminal domain of TIMP-1 effectively inhibits the enzymatic activity of MMP-2, but not of MT1-MMP. These paradoxical roles of TIMP-1 in both activating an MMP cascade and inhibiting MMP activity are likely to be modulated by the availability of the TIMP-1 binding cell surface proteins such as CD63 as well as the localization of TIMP-1 (soluble *vs*. pericellular). While the N-terminal MMP inhibitory domain of TIMP-1 interacts with and inhibits active MMPs such as MMP-2 and MMP-9, the C-terminal domain of TIMP-1 binds to pro-MMP-9. Thus, it is plausible that pro-MMP-9 and CD63 may compete for binding to the C-terminal domain of TIMP-1, and thereby pro-MMP-9 abrogates TIMP-1′s signaling capacity. This may provide an explanation for unexpected, inverse correlation between MMP-9 expression and tumor promotion. [42]. Similarly, downregulation of MMP-9 expression in human HT1080 fibrosarcoma cells increased intravasation and lung metastasis in the chick embryo chorioallantoic membrane model [43]. In the present study, we found that TIMP-1 signaling downregulates MMP-9 expression. Our model predicts that TIMP-1 molecules, which are free of pro-MMP-9, would effectively interact with its cell surface binding partner for cell signaling. Thus, TIMP-1′s ability to downregulate MMP-9 expression through TIMP-1 mediated cell signaling may indirectly promote TIMP-1′s signaling capacity, resulting in a positive feedback amplification of TIMP-1 signaling.

The present study demonstrated that TIMP-1 signaling upregulates several EMT transcription factors and MT1-MMP expression. This is of particular importance in view that MT1-MMP is among the triad of MMPs (MT1- MT2- and MT3- MMPs), essential for invasive behavior of tumor cells [44]. Moreover, the relevance of MT1-MMP to human cancers is best demonstrated, as it is suggested as a biomarker or as a potential tumor target [45], [46], [47], [48], [49], [50]. Consistent with our finding, previous studies showed that increased MT1-MMP expression promotes the EMT-like properties including cell invasion, which is associated with increased EMT transcription factors, such as Snail, Slug, and Sip1 [10], [51], [52], [53]. Zeb1 and Twist are more commonly expressed in metastatic tumors compared to the primary tumors [54] and Twist may function upstream of Snail and Zeb1 [55]. Unique and overlapping roles of these transcription factors for the regulation of epithelial/mesenchymal marker expression as well as MMPs during the TIMP-1-mediated EMT process remain to be fully investigated.

Collectively, our findings suggest a model in which TIMP-1 functions as signaling molecule and also as an endogenous inhibitor of MMPs. Thus, in spite of the fact that TIMP-1, as opposed to TIMP-2, does not interact with and inhibit MT1-MMP, TIMP-1 may act as a key regulator of MT1-MMP/MMP-2 axis. This concept represents a paradigm shift in the current view of TIMP-1/MT1-MMP interactions and functions during cancer development/progression. This information may also be useful in designing more rational, mechanism-based therapeutic interventions aimed at modulating activities of MMPs and TIMPs.
